# Evaluation of Pharmacovigilance System in Iran

**DOI:** 10.34172/ijhpm.2020.243

**Published:** 2020-12-14

**Authors:** Malahat Khalili, Hamid Sharifi, Bita Mesgarpour, Mehrnaz Kheirandish, Sten Olsson, Naghmeh Javidnikou, Ali Akbar Haghdoost

**Affiliations:** ^1^HIV/STI Surveillance Research Center, and WHO Collaborating Center for HIV Surveillance, Institute for Futures Studies in Health, Kerman University of Medical Sciences, Kerman, Iran.; ^2^National Institute for Medical Research Development, Tehran, Iran.; ^3^Department for Assessment and Control of Prescribing and Use of Medicines and Health Products, Food and Drug Administration, Tehran, Iran.; ^4^International Society of Pharmacovigilance, London, UK.; ^5^Pharmacovigilance Consulting, Uppsala, Sweden.

**Keywords:** Pharmacovigilance, Adverse Drug Reaction, Pharmacoepidemiology, Iran

## Abstract

**Background:** Evaluating a pharmacovigilance system helps identify its deficiencies and could facilitate measures to remedy and improve the quantity and quality of adverse drug reaction (ADR) reports and other opportunities for pharmacovigilance systems strengthening. This study aimed to evaluate the status of pharmacovigilance in Iran using the World Health Organization (WHO) pharmacovigilance indicators with the prospect of identifying the gaps and areas for improvement.

**Methods:** This study was conducted in 2 parts. The first part included a secondary analysis of the national data obtained from the Iranian National Pharmacovigilance Center (PVC) using a structured data collection form based on WHO core pharmacovigilance indicators. In the second part, a 3-month prospective study was carried out to investigate 2 outcome indicators, ie, length of stay and costs of medicine-related hospitalization in all patients of 2 main referral hospitals in the southeast and north of Iran.

**Results:** Iran has a PVC with national policy, trained staff, and a statutory budget. In 2017, the number of ADR reports was 15.0 per 100 000 population, and 262 signals were detected during the preceding 5 years. The average length of stay and costs of medicine-related hospitalization were 5 days and US$817.2 in Afzalipour hospital and 6.6 days and US$306.7 in Razi hospital, respectively. The status of pharmacovigilance in the Iranian public health programs (PHPs) is unknown, and most of the indicators could not be assessed.

**Conclusion:** A robust pharmacovigilance system is a pivotal part of the overall medicines regulatory system. The Iranian pharmacovigilance system has relatively the proper structural condition. Though the underreporting of ADRs, especially medicine-related deaths, is an important issue, and some indicators’ status was unclear. The Iranian pharmacovigilance program requires a higher prioritization, particularly in the PHPs, a greater allocation of resources, and cross-sectoral cooperation to bolster and achieve the pharmacovigilance objectives.

## Background

**Key Messages**

** Implications for policy makers**

- Despite a basic pharmacovigilance structure, resource, policy, and regulatory framework, the Iranian pharmacovigilance program needs suitable and sustained improvement.
- The Iranian pharmacovigilance program requires a higher prioritization of pharmacovigilance in its public health programs (PHPs) and the greater allocation of resources to bolster and achieve the pharmacovigilance objectives.
- The suitable and sustained promotion of the pharmacovigilance program can be facilitated by improved collaboration with professional organizations, including participation in educational events and scientific meetings.

** Implications for the public**

An effective pharmacovigilance system ensures the monitoring of medicines, their availability, and safe use. Therefore, it is essential to evaluate the measurement, monitoring, and effectiveness of pharmacovigilance systems, including an estimation of their impact on society. The results of the present study revealed that pharmacovigilance has a relatively satisfactory status in Iran in terms of some of the World Health Organization (WHO) pharmacovigilance indicators. The underreporting of adverse drug reactions (ADRs), especially medicine-related deaths, is an important issue that still requires attention. Furthermore, the increased length of hospital stay and costs of medicine-related hospitalization in patients who developed an ADR highlighted the importance of promotion of the pharmacovigilance system and medication safety.

Medicine utilization and expenditures have increased dramatically in the past 3 decades.^1-3^ Besides the therapeutic benefits that justify their use, medicines can also induce unwanted effects.^4^ Pharmacovigilance is a fundamental tool in clinical medicine and public health to minimize the adverse outcomes of using medicines.^3,5^ WHO defines pharmacovigilance as “the science and activities related to the detection, assessment, understanding, and prevention of adverse drug effects or any other possible drug-related problems.”^6^

 Pharmacovigilance activities identify suspected adverse drug reactions (ADRs) and evaluate the effectiveness of medicines in real-world situations, ensure patients’ safety, decrease mortality and morbidity associated with adverse reactions and promote the rational use of drugs.^5,7,8^ By communicating the risks and benefits, pharmacovigilance data support decision-making about medicines at various levels of the healthcare system,^9^ and provide information and knowledge informing regulatory actions such as drug safety alerts, product label changes, and drug recalls or withdrawals from the market.^10^ Spontaneous ADR reporting to local and national regulatory authorities is a widely-used method of pharmacovigilance.^11-14^

 The World Health Organization (WHO) supports countries in promoting sustainable monitoring systems under its “*Program for International Drug Monitoring.*”^2,10,15^ The WHO’s regional offices support the implementation of this program in low- and middle-income countries to respond to their needs while taking into account the intended state-of-the-art pharmacovigilance initiatives.^16^ Issues related to drug use and adverse event profiles can vary from one country to another due to differences between manufacturing processes, local therapeutic practices, and the population’s genetic factors. Therefore, every country should develop its national pharmacovigilance system.^17,18^

 Evaluating a pharmacovigilance system will facilitate actions to remedy the identified deficiencies to improve ADR reports’ quantity and quality, leading to more rigorous decision-making in pharmacovigilance.^19^ Currently, the assessment of pharmacovigilance has mainly been carried out at the national level using different tools, including the WHO Minimum Requirements for a Functional Pharmacovigilance System,^5^ and the Indicator based Pharmacovigilance Assessment Tool.^20^ WHO pharmacovigilance indicators were introduced in 2015. These indicators are based on the expected structures, functions, and performance of pharmacovigilance systems as described in the WHO’s minimum requirements for a functional pharmacovigilance system.^15^

 The Pharmacovigilance Center (PVC) of Iran began its activities under the supervision of the Iran Food and Drug Administration (Iran FDA) in 1991. It then became a full member of the WHO International Drug Monitoring Program in 1998. In this nationwide system, ADR reporting is voluntary through the yellow card scheme. In Iran, most previous research on pharmacovigilance has dealt with assessments of the knowledge, attitude, and practice concerning ADR,^21-25^ ADR occurrence,^26-29^ and the promotion of ADR reporting.^30-32^ In the first 10 years of the PVC activity, the under-reporting of ADR was a common drawback,^33^ but in recent years, ADR reporting has witnessed a growing trend.^34^ In Iran, the main causes of under-reporting are unawareness about the existence of a national PVC,^35,36^ inadequate knowledge regarding reporting,^37,38^ and fear of punishment and criticism.^37^ This system’s status and efficiency are presently unknown, and there is little systematic and adequate data on the effectiveness and functionality of the Iranian PVC. Therefore, this study intends to assess the status of the pharmacovigilance structure, processes, and outcomes in Iran using WHO pharmacovigilance indicators with the prospect of identifying the gaps and the most urgent pharmacovigilance priorities. The results of this assessment can help define the elements of a sustainable pharmacovigilance strategy and areas for improvements and thus provide the basis for a plan to improve public health and safety concerning the use of medicines.

## Methods

###  Study Setting and Design 

 This study was conducted in 2 parts in 2019. The first part included secondary analysis of the national data obtained from the PVC. The second part included a 3-month prospective study in 2 main referral hospitals in the southeast and northern parts of Iran. Table 1 summarizes overall the demographics, the healthcare system, and pharmaceutical characteristics in Iran.

**Table 1:** Demographics, the Healthcare System, and Pharmaceutical Characteristics in Iran

**Total population (2016 census) **	79 926 270
**Male:female**	1.03
**Life expectancy (y)**	76.27
**Mean of age (y)**	31.10
**Age dependency ratio (y)**	43.44
**Total number of importer and manufacturing pharmaceutical companies, 2017**	298
**Total number of registered drugs, 2017**	3360
**Total number of medicines in the national list of essential medicines, 2017**	402
**Hospital beds, 2016**	117 580
**Total number of health professionals, 2016**	
**Specialists**	36 345
**General practitioners**	42 188
**Pharmacists**	13237
**Dentists**	17 806
**Nurses**	106 465

###  Part 1: The Secondary Analysis of the National Data

 We conducted a secondary analysis using national data from the Iranian PVC in 2017. The coreWHO pharmacovigilance indicators were used to evaluate the status of the Iranian national pharmacovigilance program. Ten qualitative structural indicators were used to assess the existence of key pharmacovigilance structures, systems and mechanisms. The existence of a policy and regulatory framework that enables the functioning of pharmacovigilance was also assessed. The extent of the pharmacovigilance activities was measured using 9 process indicators focused on the set of activities that describe the mechanism of pharmacovigilance, ie, the collection, collation, analysis and evaluation of ADR reports. The output and outcome of pharmacovigilance activities were also measured using 8 outcome and impact indicators. Moreover, the implementation and effectiveness of pharmacovigilance within public health programs (PHPs) (eg, immunizations, infectious and zoonotic disease surveillance, non-communicable disease prevention and treatment, mental health and addiction, animal bites, disaster and injury prevention, and family planning program) were monitored and evaluated by 9 indicators.^15^ Data were obtained on the indicators from the following sources (see Figure):

**Figure:**
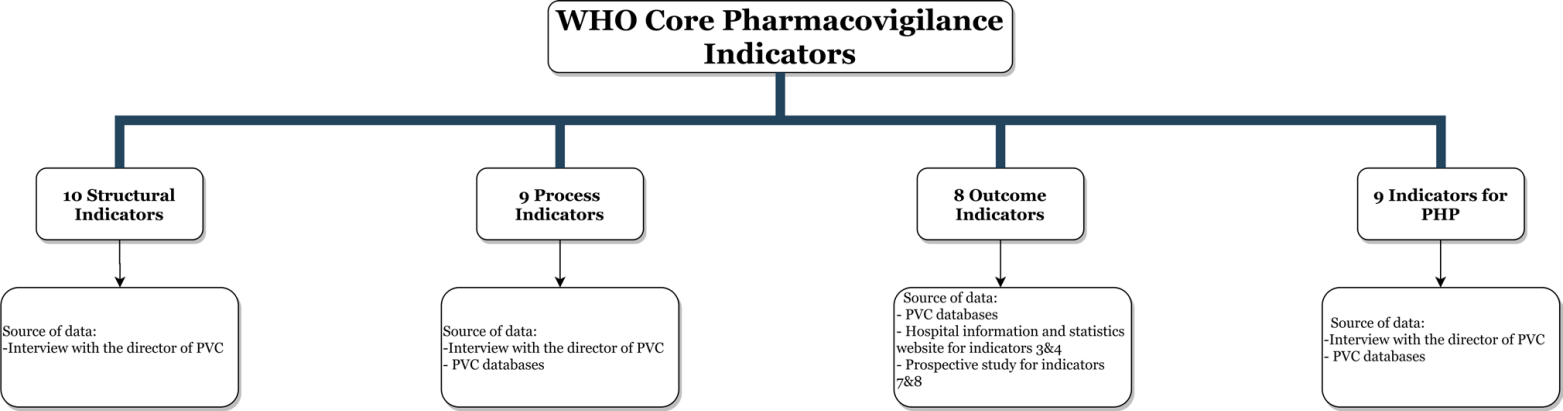
WHO Core Pharmacovigilance Indicators and Source of Data. Abbreviations: WHO, World Health Organization; PVC, Pharmacovigilance Center; PHP, public health program.

####  a. National Pharmacovigilance Center and PVC Databases

 Data were obtained through interviews with the director of the PVC using a structured data collection form based on the WHO pharmacovigilance indicators. This form involved 4 elements: Structural indicators, process indicators, outcome/impact indicators, and indicators for the PHPs. The data collection form can be found in Supplementary file 1.

####  b. The Hospital Information and Statistics Website of the Iranian Ministry of Health 

 The total number of inpatients and outpatients of hospitals throughout the country in 2017 was obtained from the hospital information and statistics website to calculate 2 outcome/impact indicators: “*Number of medicine-related hospital admissions per 100 000 admissions*” and “*Number of medicine-related deaths per 100 000 people served by the hospital.*”

###  Part 2: A Prospective Study in 2 Tertiary Hospitals in Kerman and Rasht Cities

 For calculating 2 outcome/impact indicators (*average duration of medicine-related extension of hospital stay and the average cost of medicine-related hospitalization*), a 3-month prospective observational study was carried out in Afzalipour hospital (January to March 2019) in the southeast and Razi hospital (August to October 2019) in the north of Iran. The hospitals were selected based on the level of medical care, variety in medical wards, hospital size, different location, and their accessibility and collaboration.

 Afzalipour teaching hospital is the main referral hospital in Kerman, a city in the southeast of Iran, with 21 inpatient and 3 outpatient wards (eg, pediatric, internal, surgical, gynecology, intensive care units, cardiac care units, and emergency units, etc). This hospital has 370 active beds and an 80% occupancy rate. It has 1123 employees, and around 300 students are trained in this center every year. Razi hospital is the largest teaching hospital in Rasht, a city in northern Iran, and has 240 active beds. This hospital provides the most diverse services, such as poisoning, internal, surgical and intensive care, and specialized care.

 All the patients admitted to both hospitals who had developed a suspected ADR after admission or admitted primarily for the treatment of an ADR were included in the study, except for the psychosomatic, transplant, hematology, and oncology patients. A trained epidemiologist identified all the suspected ADRs through daily visits at the hospital wards and by soliciting information from the physicians and nurses and interviewing the affected patients. The WHO definition of ADR was used in this research,^39^ and all the identified suspected ADR cases were reviewed and confirmed by a clinical pharmacist, according to WHO-the Uppsala Monitoring Centre (WHO-UMC) causality assessment system.^40^ The participants verbally consented to take part in the research. The names of the respondents were not recorded, and reassurance was given that the information would be kept confidential. The control group consisted of all the patients in the study period who were not admitted due to an ADR and had not developed a suspected ADR after admission, except for the psychosomatic, transplant, hematology, and oncology patients.

 Data were recorded using a structured ADR reporting form. For the suspected ADR-patients, data about admission date and ADR event date were collected through interviews with the patients and reviewing their medical records. Furthermore, the discharge date and hospital costs (hoteling, clinical treatment and para-clinical services, prescribed drugs, and medical equipment used in the ward) were extracted from the hospital information system database. For all the non-ADR patients (ie, the control group), data about admission date, discharge date, and hospital costs were retrieved from the hospital information system database.

###  Data Analysis

 The ten structural indicators were qualitative, and the presence or absence of the parameter measured was described. The 9 process and 8 outcome indicators were quantitative and reflected the absolute numbers, percentage, and rates of reports as determined by the indicator. The difference between the mean length of stay between the patients with and without ADR made up the estimated excess length of stay. The difference between the mean total hospital costs in the patients with ADR and the mean total hospital costs in the patients without ADR made up the estimated extra cost. Data were descriptively analyzed using Microsoft Excel 2013 software (Microsoft, Redmond, WA, USA).

## Results

 The findings from the assessment were reviewed, taking into consideration the 4 pharmacovigilance components. The results were used for each component to determine how the Iranian PVC measures up to a fully functional pharmacovigilance system.

###  Core Structural Indicators

 The PVC had a national policy and legislation enacted by the government to support pharmacovigilance activities. Moreover, the Department of Assessment and Control on Prescribing and Use of Medicines and Health-related Products, Iran FDA, is the focal point for promoting Iran’s pharmacovigilance. The PVC has trained staff and a statutory budget to properly carry out pharmacovigilance functions at regional and national levels. Nonetheless, the system suffers from a shortage of human resources. ADR reporting forms are available in governmental and private medical centers, but the form does not include sections to allow practitioners to report all the domains covered by pharmacovigilance. The Iranian national pharmacovigilance system handles ADR reports using a chain of activities, such as collection, recording, causality assessment, feedback, and submission to the WHO. Moreover, pharmacovigilance is not in the national curriculum of various healthcare professions (Table 2).

**Table 2:** Analysis of WHO Core Pharmacovigilance Structural Indicators in Iran

**Assessment Indicators**	**Answers**	**Description**
1. Existence of a pharmacovigilance center, department, or unit with a standard accommodation	Yes	Center for the registration and reporting of health products' safety and adverse effect, with non-standard accommodation (a small office space, the shortage in some basic office equipment and facilities required to receive, analyze and transmit ADR reports).
2. Existence of a statutory provision (national policy, legislation) for pharmacovigilance	Yes	- Guidelines for registration of ADR and medication errors reporting with the signature of the Minister of Health (available at: https://www.fda.gov.ir/en): as a mandatory policy in the Iranian FDA. - Legal commission vote on manufacturing and import of medicines in 2005.
3. Existence of a medicines regulatory authority or agency	Yes	Department of Assessment and Control on Prescribing and Use of Medicines and Health-related Products.
4. Existence of any regular financial provision (eg, statutory budget) for the pharmacovigilance center	Yes	The annual budget for interventions and activities such as education about ADR reporting and pharmacovigilance, holding workshops and training courses, implementing related projects, and empowering regional pharmacovigilance centers.
5. Existence of human resources to carry out its functions properly for the pharmacovigilance center	Yes	There was a shortage of human resources (only 5 pharmacists and physicians).
6. Existence of a standard ADR reporting form in the setting	Yes	Yellow ADR form and online system for ADR and medication error reporting: https://adr.ttac.ir
6a. The standard reporting form provides for reporting: suspected medication errors, suspected counterfeit/substandard medicines, therapeutic ineffectiveness, suspected misuse, abuse of and/or dependence on medicines, ADRs by the general public	No	Only the Yellow ADR form is available, and all drug-related problems are reported by this form. Moreover, the general population could report ADR via website: https://adr.ttac.ir
7. Existence of a process in place for collection, recording, and analysis of ADR reports	Yes	Reports of suspected ADRs submitted voluntarily to a regional center or the national regulatory authority by healthcare professionals or patients via completing the yellow card, e-mail, telephone, fax, or online website. Finally, all reports are assessed and analyzed by the staff of PVC.
8. Incorporation of pharmacovigilance into the national curriculum of the various healthcare professions (medical doctors, dentists, pharmacists, nurses or midwives, and others)	No	Pharmacovigilancehas not been incorporated into the national curriculum of the various healthcare professions.
9. Existence of a newsletter, information bulletin and/or website as a tool for dissemination of information on pharmacovigilance	Yes	Dissemination of information via https://www.fda.gov.ir/en
10. Existence of a national ADR or pharmacovigilance advisory committee or an expert committee in the setting capable of providing advice on medicine safety	Yes	Predominantly physicians and pharmacists serving as members of this committee and have 5 main members. They hold their meetings occasionally.

Abbreviations: WHO, World Health Organization; ADR, adverse drug reaction; MoH, Ministry of Health; PVC, Pharmacovigilance Center.

###  Core Process Indicators

 A total of 11 968 ADR reports were received in 2017 (15.0 per 100 000 population) by the PVC, and the total number of reports from 2004 to 2017 was 78 292. Only 8.2% of the reporters received some individual acknowledgment and feedback from the PVC. All the reports were satisfactorily completed and submitted to the PVC; the PVC staff assesses all submitted reports. Initial incomplete and erroneous reports are followed up by contacting reporters and finally completing. After removing duplicate and erroneous reports and reports without a causal relationship between an adverse reaction and a drug, only 43.8% of them were submitted to the WHO database. A limited number of reports were on medication errors and therapeutic ineffectiveness. Furthermore, all pharmaceutical companies (importer and national manufacturing) are required to have a functional pharmacovigilance system (Table 3).

**Table 3:** Analysis of WHO Core Pharmacovigilance Process and Outcome Indicators of Iran

**Assessment Indicators**	**Answers**	**Description**
**Core Process Indicators**		
1- Total number of ADR reports received as number per 100 000 population in 2017	15.0	11 968 ADR reports in 79 926 270 inhabitants.
2- Current total number of reports in the national, regional, or local database	78 292	Number of ADR reports from 2004 to 2017.
3- Percentage of total annual reports acknowledged/issued feedback in 2017	8.2%	All severe reports, reports of an increase in signal frequency, and reports from scientific associations and other MoH departments are given feedback (n = 985).
4- Percentage of total reports subjected to causality assessment in 2017	100%	All received reports are subjected to causality assessment by WHO-UMC criteria.
5- Percentage of total annual reports satisfactorily completed and submitted to the national pharmacovigilance center in 2017	100%	Reports satisfactorily have filled all the relevant fields for causality assessment.
5a- Percentage of the reports satisfactorily completed and submitted to the national pharmacovigilance center, the percentage of reports committed to the WHO database	43.8%	5236 ADR reports.
6- Percentage of reports of therapeutic ineffectiveness received in 2017	2.4%	286 reports.
7- Percentage of reports on medication errors reported in 2017	6.2%	745 reports.
8- Percentage of registered pharmaceutical companies having a functional pharmacovigilance system	100%	All registered pharmaceutical companies (manufacturers, distributors, and importers) (n = 298) are required to set up the pharmacovigilance system. Iranian PVC is responsible for monitoring the performance of this system in pharmaceutical companies.
9- Number of active surveillance activities initiated, ongoing or completed in 2013-2017	24	Activities to promote the safety of medicines and health products.
Core Outcome Indicators		
1- Number of signals detected in 2013-2017 by the pharmacovigilance center	262	Signal detection is done by the WHO signal detection method for serious or unexpected reports, unexpected increases in the frequency of reports of adverse reactions, or increased reports of drugs' therapeutic ineffectiveness. A qualitative evaluation of drugs is done by the Food and Drug Control Reference Laboratories for confirmation in most cases.
2- Number of regulatory actions taken consequent to national pharmacovigilance activities in 2017	136	Medication safety warnings and notices disseminate to healthcare professionals and the public. Based on the received reports, the label changes were suggested to the Iranian FDA.
- Product label changes (variation)	8	
- Safety warnings on medicines	117	
- Drug withdrawals	0	
- Other restrictions on the use of medicines	11	
3- Number of medicine-related hospital admissions per 100 000 admissions in 2017	Unavailable data	There was not a link between the deputy for curative affairs and PVC to get this information.
4- Number of medicine-related deaths per 100 000 people served by the hospital in 2017	Unavailable data	
5- Number of medicine-related deaths per 100 000 persons in the population in 2017	0.09	71 medicine-related mortality in the population-based on spontaneous reporting.
6- Average cost (US$) of treatment of medicine-related illness	Inadequate data	Due to the lack of adequate data, this indicator was not calculated.
7- Average duration (days) of medicine-related extension of hospital stay	Afzalipour hospital: 5.0 days Razi hospital: 6.6 days	At the tertiary hospitals level, in Kerman and Rasht cities.
8- Average cost (US$) of medicine-related hospitalization	Afzalipour hospital: US$817.2 (IRR 103 791 328) Razi hospital: US$306.7 (IRR 38 956 290)	

Abbreviations: WHO, World Health Organization; UMC, Uppsala Monitoring Centre; ADR, adverse drug reaction; MoH, Ministry of Health; PVC, pharmacovigilance center; IRR, Iranian Rial; US$, United States Dollar; FDA, Food and Drug Administration.

###  Core Outcome/Impact Indicators 

 As Table 3 shows, the PVC of Iran detected 262 signals to indicate a possible causal relationship between an adverse event and a drug (including chemical and conventional medicines, herbal medicines, other traditional and complementary products, and biological and blood products) during the examination period of the 5 years. Moreover, 136 regulatory actions (such as product label changes, safety warnings on medicines, drug withdrawals, and other restrictions on the use of medications) had been taken in 2017. Furthermore, there were 71 spontaneous medicine-related deaths in the population.

 According to the primary study results, 69 ADR cases were detected in the 8739 admissions (789.6 per 100 000 admission)

 at Afzalipour hospital and 46 cases in the 4885 admissions (941.7 per 100 000 admission) at Razi hospital. The costs of hospitalization necessitated by a medicine-related illness and the in-hospital medicine-related disease incidence were estimated at US$817.2 and US$306.7 in Afzalipour and Razi hospitals, respectively. The duration of hospital stay in these patients was almost double that of the other patients (Table 3).

###  Pharmacovigilance Indicators for Public Health Programs

 Iranian PVC does not systematically gather ADR reports from the Iranian PHP because there is not an in charge of ADR reporting in the PHP (Table 4). Consequently, the status of pharmacovigilance in PHP is unknown, and most of the indicators could not be assessed and calculated due to insufficient information. However, 235 ADR reports had been collected within PHP, such as vaccination, tuberculosis, leprosy, and HIV/AIDS, and all of them were submitted to the WHO database in 2017.

**Table 4:** Analysis of WHO Pharmacovigilance indicators for Public Health Programs of Iran

**Assessment Indicators**	**Answers**	**Description**
1- Pharmacovigilance activities in place within the PHP	Yes	Correspondence with the health deputy about reporting of ADR and medication error was done by PVC.
2- All main treatment guidelines and protocols in use within the PHP systematically consider Pharmacovigilance	No	Protocols and guidelines of PHP are under the supervision of the deputy for health and do not routinely have pharmacovigilance and ADR reporting section.
3- Existence of standard ADR reporting form in the setting	Unknown	The ADR form is not as the main form in the PHP and for health deputy.
- The standard reporting form provides for reporting: suspected medication errors, suspected counterfeit/substandard medicines, therapeutic ineffectiveness, suspected misuse, abuse of and/or dependence on medicines	Unknown	
4- Total number of ADR reports collected within the PHP in 2017	235	These reports had been received from PHP, such as vaccination, tuberculosis, leprosy, and HIV/AIDS.
5- Total number of ADR reports per 1000 individuals exposed to medicines in the PHP in 2017	Inadequate data	The number of individuals exposed to medicines in the PHP was unknown.
6- Total number of reports on therapeutic ineffectiveness in 2017	Unknown	The number of reports on medicine and vaccines' ineffectiveness was unknown.
7- Percentage of completed reports submitted to the national pharmacovigilance center in 2017	100%	The number of completed reports were unknown.
- Of the reports satisfactorily completed and submitted to the national pharmacovigilance center, the percentage of reports committed to the WHO database	100%	All reports that received from PHP committed to the WHO database.
8- Number of medicine-related hospital admissions per 1000 individuals exposed to medicines in the PHP in 2017	Unknown	The number of medicine-related hospital admissions was unknown.
9- Number of medicine-related deaths per 1000 individuals exposed to medicines in the PHP in 2017	Unknown	The number of medicine-related deaths was unknown.

Abbreviations: WHO, World Health Organization; ADR, adverse drug reaction; PVC, Pharmacovigilance Center; PHP, public health program.

## Discussion

 To the researchers’ knowledge, the present study is the first systematic assessment of the pharmacovigilance program in Iran using the core WHO pharmacovigilance indicators. Despite the existence of a functioning pharmacovigilance structure and resource, policy and regulatory framework, the performance and achievements of the Iranian pharmacovigilance program need suitable and sustained improvement. The state of completeness and causality assessment of the reports was satisfactory and the PVC of Iran had appropriately utilized statistical methods to help detect signals from the ADR reports. The underreporting of ADR per population and medicine-related deaths was the drawback of this system. Moreover, the status of medicine-related hospital admissions and deaths and also pharmacovigilanceactivities in the Iranian PHP could not be studied under the prevailing circumstances. The estimated duration of hospital stay and cost of medicine-related hospitalization were found to be relatively high in the primary study at the hospital level.

 The status of structural indicators in Iran’s pharmacovigilance program has demonstrated a relatively satisfactory commitment to improving medicine safety and providing direction to enhance the system. This program had a statutory budget and limited human resources to function. The absence of pharmacovigilance in the training curriculum for healthcare professionals in Iran suggests their lack of preparedness for career challenges on medicine safety issues. It should be noted that pharmacovigilance activities are one of the National Hospital Accreditation Program standards in Iran, and it is routinely performed and checked since 2016.^41^ A study by Qato et al showed that most Arab and Eastern Mediterranean countries had pharmacovigilance programs, and the majority were government-funded, but staff resources were limited.^2^ Moreover, in the study by Olsson et al on 55 low- and middle-income countries, few countries had any budget allocated for pharmacovigilance, and most centers were inadequately staffed. In most of them, pharmacovigilance was absent from the training curriculum of the healthcare professions.^16^ At present, developed and some developing countries are incorporating pharmacovigilance into the curriculum of health disciplines.^7,42-46^

 Regulations, structures, frameworks, policies, and roles provide a foundational basis for the organized and systematic operationalization of pharmacovigilance activities that will enable the effective and efficient use of staff, skills, and tools.^47^ When these provisions are matched with a regular and sustainable budget, real action and long-term planning can be achieved.^48^ Despite the existence of legislation and guidelines for registration of ADR in Iran, there is no clear structure of legal requirements to ensure regulatory compliance. Moreover, the presence of highly-qualified pharmacovigilance professionals and the capacity to employ them is important for developing a robust pharmacovigilance system. Proper training and the introduction of pharmacovigilance concepts in healthcare professions’ academic education or job curriculums can also help build knowledge and raise awareness about ADRs.^49,50^

 As for the process indicators, the ADR rate was 15.0 per 100 000 of the population in 2017. This rate is low in Iran compared to many developed countries,^51-56^ with rates ranging from 17.0 to 323.0 per 100 000 population. Nonetheless, the rate was higher in Iran than in Turkey,^57^ Ethiopia,^58^ Kenya, and Tanzania,^59^ which was 0.4 to 3.5 per 100 000 population. Furthermore, medicine-related death is a measure of the harmful effects of medicines in the community on hospitalized or non-hospitalized patients.^15^ In Iran, the rate of medicine-related deaths based on spontaneous reporting was 0.09 per 100 000 population, while it was 0.12 per 100 000 population in the United States.^60^ Underreporting is a limitation of the Iranian pharmacovigilance system due to the passiveness of the system. Given the growing trend of reporting in recent years in Iran, the reporting rate is expected to rise in the near future.^34^ The low feedback rates in the Iranian pharmacovigilance system may discourage the reporting of ADRs by HCP. While general feedback to ADR reporters and a positive, active relationship between the HCP and the PVC could stimulate ADR reporting in HCP. Sending feedback to the HCP could also increase the knowledge and awareness about ADR reporting.^61^ As observed in other countries,^62-65^ registered pharmaceutical companies in Iran have a functional pharmacovigilance system and an effective reporting system. As well as, they develop and submit periodic safety update reports to appropriate authorities. Certain stringent regulations are required to oblige pharmaceutical companies, particularly local and generic companies, to involve themselves in pharmacovigilance activities.^48^

 Concerning the outcome indicator measures, 262 signals were detected during the 5 years. Over 100 signals were also detected in Canada, the United States, and Switzerland during 5 years^66^; as well as it was 239 for European Union during 4 years.^67^ Thirty-three percent of the Association of Southeast Asian Nations countries^66^ and 85% of Arab and Eastern Mediterranean countries^2^ utilized statistical methods to help detect signals from the ADR reports. The pharmacovigilance system’s ability to detect signals underscores its relevance in identifying safety problems and promoting the safe use of medicines.^15^ Moreover, PVC has actively taken regulatory decisions on medicines to protect public health. There was an extensive range of these activities in previous studies.^2,9,16,68^ In a study on 55 low- and middle-income countries, some countries took no action, while others reported more than 3 market withdrawals over a year.^16^ Singapore reported 229 label changes, 23 product safety alerts, 6 product recalls, and 29 letters to healthcare professionals in 2011.^9^ Since there are vast differences between countries’ pharmaceutical market and legal systems, no judgment can be made whether pharmacovigilance data have been under or over-utilized in countries for regulatory decision-making.

 Although an accurate assessment of ADR’s financial costs is difficult, a crude estimate suggests that the total costs are likely increased in ADR patients compared to non-ADR patients. The estimated costs were remarkably lower than in previous studies.^69-72^ Furthermore, ADRs were associated with an increase in the length of hospital stay. Other studies have shown that hospitalized patients experiencing an ADR extend their hospital stay from 1.9 to 9.3 days.^69,70,73-75^ Nonetheless, these differences might also be derived from the quality of the delivered medical care and methodological differences, specifically regarding adjustment for comorbidities and illness severity. It has been suggested that the occurrence of an ADR could contribute to increased hospital stays. It can also be assumed that longer hospital stays lead to the use of more drugs or the use of drugs for a longer period, and these factors would also favor the occurrence of an ADR.^71^ In any event, the prolongation of hospital stay has important medical and economic consequences. Training patients and the prescribers of medications may lessen the economic burden on patients and the healthcare system.^76,77^ Besides, Afzalipour hospital has more special wards and beds (eg, surgical intensive care unit [ICU], medical ICU, neonatal ICUs, pediatric ICU, poisoning ICU, critical care units) than Razi hospital. Therefore, a higher number of ADRs occurred in these wards, and as a result, the length of hospital stay and the cost were higher in Afzalipour hospital.

 The majority of PHPs in the health system use medicines, which represents a substantial investment in pharmaceuticals. PHPs must include a good pharmacovigilance strategy to monitor the safety and safe use of their medicines.^15^ In Iran, however, the status of pharmacovigilance activities is obscure within the PHP, and the number of reports received from the PHP is relatively small. Most of the adverse reactions of medicines and vaccines at PHP are reported to the health deputy. Since Iranian PVC does not have supervision and collaboration with the health deputy and PHP for pharmacovigilance activity and ADR reporting, ADR reports are not systematically gathered from the Iranian PHP. The situation is similar in Middle Eastern^78^ and other developing countries.^3,9,42^ The relatively small volume of ADR reports received in the PHP likely reflects most of these programs’ novelty and would be expected to grow as the systems mature. Furthermore, pharmacovigilance and ADR monitoring in PHP can detect rare adverse events and risk factors in patients and have a tremendous positive impact on the implementation and success of these programs. The PHP should also support policies to address inequities in pharmacovigilance, particularly in rural areas, which are disproportionately disadvantaged and have fewer hospitals and pharmacosurveillance units than urban centers.^8^ In Iran, further pursuits by the PVC and stronger cooperation with the health deputy could improve pharmacovigilance activity in PHP.

 There are some limitations in this study. The WHO indicators were quite useful in this assessment; however, their sensitivity and specificity as a measurement tool were not specified. Furthermore, a scoring system would be useful to quantify the indices and highlight the deficiencies in numerical terms. Another limitation of this study is that the staff of the PVC completed the data collection form without access to raw data for authors, which can lead to a biased conclusion. Furthermore, extensive information about the system could not easily be collected; thus, for 2 indicators, the results of a primary study at the hospital level were used. Meanwhile, the national-level data were used for the rest of the indicators, which means that the retrieved data were not collected primarily for research purposes and often contain incomplete, inconsistent, or wrong information. We were also unable to collect data for the PHP assessment directly from the programs as encouraged by the WHO practical manual to assess pharmacovigilance systems; these indicators had evaluated according to PVC information.

 Actions such as monitoring the implementation of rules and regulations, providing the necessary infrastructures, training in key aspects of pharmacovigilance, and developing comprehensive guidance and tools to support the best practices for the pharmacovigilance system and ADR reporting could help improve the functioning and capability of the pharmacovigilance program.^79,80^ The suitable and sustained promotion of the pharmacovigilance program can be facilitated by improved collaboration with professional organizations, including participation in educational events and scientific meetings.^78^ Furthermore, there is a great need for the introduction of pharmacovigilance for all PHPs. Moreover, active surveillance is useful for obtaining better insights into safety and tolerability patterns, especially when introducing new products to treat large populations.^15,19^ It is essential to inculcate and articulate a computerized approach to routine data gathering, documentation, and subsequent data management into the healthcare system, which facilitates the evaluation of the outcomes and impact of pharmacovigilance activities.^81^

 The WHO pharmacovigilance indicators would be useful for assessments at the regional level and tertiary hospitals, as they can help develop a strategy toward improving patient safety through pharmacovigilance. They may also help identify areas that need urgent intervention or modification in the health system. These WHO indicators could be used as a tool for quality assurance and improvement and repeated measurement of the indicators over time will allow an assessment of the progress. Awareness about regular pharmacovigilance evaluations with pharmacovigilance indicators would translate into better pharmacovigilance processes and outcomes.

## Conclusion

 In Iran, the pharmacovigilance system meets many of the WHO pharmacovigilance indicators, such as structural indicators, signal detection, causality assessment, pharmacovigilance activities in pharmaceutical companies, and regulatory decisions on medicines. The underreporting of ADRs, especially medicine-related deaths, is an important issue that needs attention. Moreover, the non-implementation of pharmacovigilance activities in the PHP, the lack of national information on the length of stay and costs of medicine-related hospitalization, the lack of information about medicine-related hospital admissions and deaths, and the limited feedback to reporters are other weaknesses of the PVC. The Iranian pharmacovigilance program requires a higher prioritization of pharmacovigilance in its PHP and the greater allocation of resources to bolster and achieve the pharmacovigilance objectives. It is crucial that everyone involved in the health system network, ranging from regulatory decision-makers to healthcare personnel, support the activities of the PVC. Moreover, cross-sectoral cooperation can significantly improve and strengthen this system.

## Acknowledgements

 We would like to express our profound sorrow at the loss of Dr. Naghmeh Javidnikou who passed away on August 14, 2020, while serving as a personnel of the MoH to fight the coronavirus disease 2019 (COVID-19) pandemic. The authors would also like to especially thank and appreciate members of the Iranian National PVC and Afzalipour and Razi hospitals’ staff for their comprehensives support for this study.

## Ethical issues

 The study protocol was approved by the Research Ethics Committee at Kerman University of Medical Sciences, Kerman, Iran (IR.KMU.REC.1397.543).

## Competing interests

 MeK reports that she has been the director-general for the Department of Assessment and Control of Prescribing and use of Medicines and Health products, Iranian Food and Drug Administration, at the time that research was completed. NJ reports that she was the head of Pharmacovigilance and Adverse Drug Reactions Office, Iranian Food and Drug Administration at the time that research was completed.

## Authors’ contributions

 MaK: Constructing the idea, methodology, data gathering, analysis and interpretation, and writing the manuscript; HS: Constructing the idea, planning, analysis and interpretation, and critical review; BM: Methodology, planning, analysis and interpretation, and writing the manuscript, critical review; MeK: Data gathering, and supervision on data gathering, interpretation of analysis, and critical review; NJ: Data gathering, analysis, and interpretation; SO: Interpretation of analysis, and critical review; AAH: Constructing the idea, supervision, planning and critical review.

## Funding

 This study was supported by Kerman University of Medical Sciences, Kerman, Iran.

## Supplementary files

Supplementary file 1. The Data Collection Form for Evaluation of Pharmacovigilance System in Iran.

## References

[1] Linnér L, Eriksson I, Persson M, Wettermark B (2020). Forecasting drug utilization and expenditure: ten years of experience in Stockholm. BMC Health Serv Res.

[2] Qato DM (2018). Current state of pharmacovigilance in the Arab and Eastern Mediterranean region: results of a 2015 survey. Int J Pharm Pract.

[3] Kabore L, Millet P, Fofana S, Berdai D, Adam C, Haramburu F (2013). Pharmacovigilance systems in developing countries: an evaluative case study in Burkina Faso. Drug Saf.

[4] Amin S, Mishra V, Mira D, Rajesh S (2018). Pattern of adverse drug reactions and its potential impact on drug resistant tuberculosis patients at a tertiary care teaching hospital in Western India. Clin J Pharmacol Pharmacother.

[5] World Health Organization (WHO). Minimum Requirements for A Functional Pharmacovigilance System. https://apps.who.int/medicinedocs/en/m/abstract/Js23393en/. Published 2010.

[6] World Health Organization (WHO). The Importance of Pharmacovigilance: Safety Monitoring of Medicinal Products. Geneva: WHO; 2002.

[7] Nwokike J, Joshi M. Pharmacovigilance in Rwanda: A Systems Analysis. https://apps.who.int/medicinedocs/en/m/abstract/Js18258en/. Published 2009.

[8] Moscou K, Kohler JC (2017). Matching safety to access: global actors and pharmacogovernance in Kenya- a case study. Global Health.

[9] Nwokike J, Ludeman E, Thumm M. Comparative Analysis of Pharmacovigilance Systems in Five Asian Countries. https://apps.who.int/medicinedocs/en/m/abstract/Js21335en/. Published 2013.

[10] Maigetter K, Pollock AM, Kadam A, Ward K, Weiss MG (2015). Pharmacovigilance in India, Uganda and South Africa with reference to WHO’s minimum requirements. Int J Health Policy Manag.

[11] Bandekar MS, Anwikar SR, Kshirsagar NA (2010). Quality check of spontaneous adverse drug reaction reporting forms of different countries. Pharmacoepidemiol Drug Saf.

[12] Arksey H, O’Malley L (2005). Scoping studies: towards a methodological framework. Int J Soc Res Methodol.

[13] Anton C, Cox AR, Ferner RE (2009). Improving follow-up rates in spontaneous adverse drug reaction reporting: effectiveness of a targeted letter used by a regional centre in the UK. Drug Saf.

[14] Chang F, Xi Y, Zhao J, Zhang X, Lu Y (2017). A time series analysis of the effects of financial incentives and mandatory clinical applications as interventions to improve spontaneous adverse drug reaction reporting by hospital medical staff in China. J Eval Clin Pract.

[15] World Health Organization (WHO). WHO Pharmacovigilance Indicators: A Practical Manual for the Assessment of Pharmacovigilance Systems. WHO; 2015.

[16] Olsson S, Pal SN, Stergachis A, Couper M (2010). Pharmacovigilance activities in 55 low-and middle-income countries: a questionnaire-based analysis. Drug Saf.

[17] Pirmohamed M, Atuah KN, Dodoo AN, Winstanley P (2007). Pharmacovigilance in developing countries. BMJ.

[18] Hoffmann E, Fouretier A, Vergne C, Bertram D (2012). Pharmacovigilance regulatory requirements in Latin America. Pharmaceut Med.

[19] Opadeyi AO, Fourrier-Réglat A, Isah AO (2018). Assessment of the state of pharmacovigilance in the South-South zone of Nigeria using WHO pharmacovigilance indicators. BMC Pharmacol Toxicol.

[20] Strengthening Pharmaceutical Systems (SPS) Program. Indicator-Based Pharmacovigilance Assessment Tool: Manual for Conducting Assessments in Developing Countries. https://apps.who.int/medicinedocs/en/m/abstract/Js21303en/. Accessed March 31, 2020. Published 2009.

[21] Vessal G, Mardani Z, Mollai M (2009). Knowledge, attitudes, and perceptions of pharmacists to adverse drug reaction reporting in Iran. Pharm World Sci.

[22] Hamedivafa F, Peiravian F (2012). A survey of knowledge, attitude and practice of nurses towards pharmacovigilance in teaching hospital, Qazvin-Iran. Res Pharm Sci.

[23] Mirzaei Alavijeh M, Karami Matin B, Mahboubi M, Jalilian F (2016). Factors related with adverse drug reaction reporting: a cross-sectional study among pharmacists in the West of Iran. Arvand J Health Med Sci.

[24] Hanafi S, Torkamandi H, Hayatshahi A, Gholami K, Shahmirzadi NA, Javadi MR (2014). An educational intervention to improve nurses’ knowledge, attitude, and practice toward reporting of adverse drug reactions. Iran J Nurs Midwifery Res.

[25] Peymani P, Tabrizi R, Afifi S (2016). Knowledge, attitude and practice of General Practitioners towards adverse drug reaction reporting in South of Iran, Shiraz (Pharmacoepidemiology report). Int J Risk Saf Med.

[26] Namazi S, Borhani-Haghighi A, Karimzadeh I (2011). Adverse reactions to antiepileptic drugs in epileptic outpatients: a cross-sectional study in Iran. Clin Neuropharmacol.

[27] Akhavan Sepahi M, Movahed Z, Heydari H, Shirkhodai M, Shokrollahi MR (2013). Surveillance of adverse drug reaction in hospitalized children, a cross sectional study from Qom province, Iran. Life Sci J.

[28] Pourseyed S, Fattahi F, Pourpak Z (2009). Adverse drug reactions in patients in an Iranian department of internal medicine. Pharmacoepidemiol Drug Saf.

[29] Mohebbi N, Shalviri G, Salarifar M, Salamzadeh J, Gholami K (2010). Adverse drug reactions induced by cardiovascular drugs in cardiovascular care unit patients. Pharmacoepidemiol Drug Saf.

[30] Baniasadi S, Namdar R, Fahimi F (2009). Development of an adverse drug reaction bulletin in a teaching hospital. Formulary.

[31] Baniasadi S, Fahimi F, Shalviri G (2008). Developing an adverse drug reaction reporting system at a teaching hospital. Basic Clin Pharmacol Toxicol.

[32] Baniasadi S, Habibi M, Haghgoo R (2014). Increasing the number of adverse drug reactions reporting: the role of clinical pharmacy residents. Iran J Pharm Res.

[33] Shalviri G, Valadkhani M, Dinarvand R (2009). Ten years pharmacovigilance activities in Iran. Iran J Public Health.

[34] Javidnikoo N, Karimi-Ghavanloo M, Kheirandish M. Report of Adverse Drug Reaction and Medication Error in 2016 and 2017. Razi Drug Monthly; 2019.

[35] Hanafi S, Torkamandi H, Hayatshahi A, Gholami K, Javadi M (2012). Knowledge, attitudes and practice of nurse regarding adverse drug reaction reporting. Iran J Nurs Midwifery Res.

[36] Etminani-Isfahani M, Mousavi S, Rakhshan A, Assarian M, Kuti L, Eslami K (2013). Adverse drug reactions: knowledge, attitude and practice of pharmacy students. J Pharm Care.

[37] Mirbaha F, Shalviri G, Yazdizadeh B, Gholami K, Majdzadeh R (2015). Perceived barriers to reporting adverse drug events in hospitals: a qualitative study using theoretical domains framework approach. Implement Sci.

[38] Afifi S, Maharloui N, Peymani P (2014). Adverse drug reactions reporting: pharmacists’ knowledge, attitude and practice in Shiraz, Iran. Int J Risk Saf Med.

[39] Edwards IR, Biriell C (1994). Harmonisation in pharmacovigilance. Drug Saf.

[40] Uppsala Monitoring Centre (UMC). The Use of the WHO-UMC System for Standardised Case Causality Assessment. https://www.who.int/medicines/areas/quality_safety/safety_efficacy/WHOcausality_assessment.pdf. Accessed March 30, 2020. Published 2018.

[41] Aghaei Hashjin A, Delgoshaei B, Kringos DS, Tabibi SJ, Manouchehri J, Klazinga NS (2015). Implementing hospital quality assurance policies in Iran: balancing licensing, annual evaluation, inspections and quality management systems. Int J Health Care Qual Assur.

[42] Allabi AC, Nwokike J (2014). A situational analysis of pharmacovigilance system in Republic of Benin. J Pharmacovigil.

[43] Marcelo J. Safety of Medicinal Products in the Philippines: Assessment of the Pharmacovigilance System and its Performance. https://apps.who.int/medicinedocs/en/m/abstract/Js22350en/. Accessed March 31, 2020. Published 2013.

[44] Cerruti L, Lebel D, Van Hees T (2015). Pilot study about hospital pharmacy residents’perception of pharmacovigilance in Belgium, France, Canada and Switzerland. J Popul Ther Clin Pharmacol.

[45] Hartman J, Härmark L, van Puijenbroek E (2017). A global view of undergraduate education in pharmacovigilance. Eur J Clin Pharmacol.

[46] Ampadu HH, Hoekman J, Arhinful D, Amoama-Dapaah M, Leufkens HGM, Dodoo ANO (2018). Organizational capacities of national pharmacovigilance centres in Africa: assessment of resource elements associated with successful and unsuccessful pharmacovigilance experiences. Global Health.

[47] Abiri OT, Johnson WCN (2019). Pharmacovigilance systems in resource-limited settings: an evaluative case study of Sierra Leone. J Pharm Policy Pract.

[48] Olsson S, Pal SN, Dodoo A (2015). Pharmacovigilance in resource-limited countries. Expert Rev Clin Pharmacol.

[49] van Eekeren R, Rolfes L, Koster AS (2018). What future healthcare professionals need to know about pharmacovigilance: introduction of the WHO PV core curriculum for university teaching with focus on clinical aspects. Drug Saf.

[50] Goel D, Farooq M (2017). Impact of educational intervention on knowledge, attitude and practice of pharmacovigilance among interns. Adv Hum Biol.

[51] Moore TJ, Furberg CD, Mattison DR, Cohen MR (2016). Completeness of serious adverse drug event reports received by the US Food and Drug Administration in 2014. Pharmacoepidemiol Drug Saf.

[52] Srba J, Descikova V, Vlcek J (2012). Adverse drug reactions: analysis of spontaneous reporting system in Europe in 2007-2009. Eur J Clin Pharmacol.

[53] Guo XJ, Ye XF, Wang XX (2015). Reporting patterns of adverse drug reactions over recent years in China: analysis from publications. Expert Opin Drug Saf.

[54] Motola D, Melis M, Lo Bianco S, Buccellato E, Biagi C, Vaccheri A (2014). Ten years of pharmacovigilance in Italy: the experience of Emilia-Romagna region in the monitoring of drug’s safety profile. Expert Opin Drug Saf.

[55] Kopečná E, Deščíková V, Vlček J, Mladá J (2011). Adverse drug reaction reporting in the Czech Republic 2005-2009. Int J Clin Pharm.

[56] Wong SX, Tham MY, Goh CL, Cheong HH, Chan SY (2019). Spontaneous cutaneous adverse drug reaction reports-an analysis of a 10-year dataset in Singapore. Pharmacol Res Perspect.

[57] Ozcan G, Aykac E, Kasap Y, Nemutlu NT, Sen E, Aydinkarahaliloglu ND (2016). Adverse drug reaction reporting pattern in Turkey: analysis of the national database in the context of the first pharmacovigilance legislation. Drugs Real World Outcomes.

[58] Ermias A, Gurmesa G, Mesfin M, Mengistu A (2011). Adverse drug reaction monitoring in Ethiopia: analysis of case reports, 2002-2007. Ethiop J Health Dev.

[59] Barry A, Olsson S, Minzi O (2020). Comparative assessment of the national pharmacovigilance systems in East Africa: Ethiopia, Kenya, Rwanda and Tanzania. Drug Saf.

[60] Shepherd G, Mohorn P, Yacoub K, May DW (2012). Adverse drug reaction deaths reported in United States vital statistics, 1999-2006. Ann Pharmacother.

[61] Oosterhuis I, van Hunsel FP, van Puijenbroek EP (2012). Expectations for feedback in adverse drug reporting by healthcare professionals in the Netherlands. Drug Saf.

[62] Vial T (2016). French pharmacovigilance: missions, organization and perspectives. Therapie.

[63] Maigetter K, Pollock AM, Kadam A, Ward K, Weiss MG (2015). Pharmacovigilance in India, Uganda and South Africa with reference to WHO’s minimum requirements. Int J Health Policy Manag.

[64] Santoro A, Genov G, Spooner A, Raine J, Arlett P (2017). Promoting and protecting public health: how the European Union pharmacovigilance system works. Drug Saf.

[65] Alshammari TM, Alshakka M, Aljadhey H (2017). Pharmacovigilance system in Saudi Arabia. Saudi Pharm J.

[66] Chan CL, Ang PS, Li SC (2017). A survey on pharmacovigilance activities in Asean and selected non-Asean countries, and the use of quantitative signal detection algorithms. Drug Saf.

[67] Farcaş A, Măhălean A, Bulik NB, Leucuta D, Mogoșan C (2018). New safety signals assessed by the Pharmacovigilance Risk Assessment Committee at EU level in 2014-2017. Expert Rev Clin Pharmacol.

[68] Suwankesawong W, Dhippayom T, Tan-Koi WC, Kongkaew C (2016). Pharmacovigilance activities in ASEAN countries. Pharmacoepidemiol Drug Saf.

[69] Hoogervorst-Schilp J, Langelaan M, Spreeuwenberg P, de Bruijne MC, Wagner C (2015). Excess length of stay and economic consequences of adverse events in Dutch hospital patients. BMC Health Serv Res.

[70] Hug BL, Keohane C, Seger DL, Yoon C, Bates DW (2012). The costs of adverse drug events in community hospitals. Jt Comm J Qual Patient Saf.

[71] Geer MI, Koul PA, Tanki SA, Shah MY (2016). Frequency, types, severity, preventability and costs of Adverse Drug Reactions at a tertiary care hospital. J Pharmacol Toxicol Methods.

[72] Natanaelsson J, Hakkarainen KM, Hägg S (2017). Direct and indirect costs for adverse drug events identified in medical records across care levels, and their distribution among payers. Res Social Adm Pharm.

[73] Poudel DR, Acharya P, Ghimire S, Dhital R, Bharati R (2017). Burden of hospitalizations related to adverse drug events in the USA: a retrospective analysis from large inpatient database. Pharmacoepidemiol Drug Saf.

[74] Khan LM (2013). Comparative epidemiology of hospital-acquired adverse drug reactions in adults and children and their impact on cost and hospital stay--a systematic review. Eur J Clin Pharmacol.

[75] Rottenkolber D, Schmiedl S, Rottenkolber M (2011). Adverse drug reactions in Germany: direct costs of internal medicine hospitalizations. Pharmacoepidemiol Drug Saf.

[76] Pattanaik S, Dhamija P, Malhotra S, Sharma N, Pandhi P (2009). Evaluation of cost of treatment of drug-related events in a tertiary care public sector hospital in Northern India: a prospective study. Br J Clin Pharmacol.

[77] Patel KJ, Kedia MS, Bajpai D, Mehta SS, Kshirsagar NA, Gogtay NJ (2007). Evaluation of the prevalence and economic burden of adverse drug reactions presenting to the medical emergency department of a tertiary referral centre: a prospective study. BMC Clin Pharmacol.

[78] Wilbur K (2013). Pharmacovigilance in the Middle East: a survey of 13 Arabic-speaking countries. Drug Saf.

[79] Bate A, Beckmann J, Dodoo A (2017). Developing a Crowdsourcing Approach and Tool for Pharmacovigilance Education Material Delivery. Drug Saf.

[80] Hans M, Gupta SK (2018). Comparative evaluation of pharmacovigilance regulation of the United States, United Kingdom, Canada, India and the need for global harmonized practices. Perspect Clin Res.

[81] Khalili M, Mesgarpour B, Sharifi H, Daneshvar Dehnavi S, Haghdoost AA (2020). Interventions to improve adverse drug reaction reporting: a scoping review. Pharmacoepidemiol Drug Saf.

