# Supplementary material for: Evaluation of Pharmacovigilance System in Iran
Source: Int J Health Policy Manag. 2020 Dec 14;11(7):990–1000. doi: 10.34172/ijhpm.2020.243 (PMC9808195; doi:10.34172/ijhpm.2020.243)
Supplement: Supplementary file 1 — The Data Collection Form for Evaluation of Pharmacovigilance System in Iran. [file ijhpm-11-990-s001.pdf]

Supplementary file 1. The Data Collection Form for Evaluation of the Pharmacovigilance System in Iran

Core structural indicators	Yes	No	Description
1- Is there a pharmacovigilance center, department, or unit with a standard accommodation?			
2- Is there a statutory provision (national policy, legislation) for pharmacovigilance?			
3- Is there a medicines regulatory authority or agency?			
4- Is there any regular financial provision (e.g., statutory budget) for the pharmacovigilance center?			
5- Have the pharmacovigilance center the human resources to carry out its functions properly?			
6- Is there a standard ADR reporting form in the setting?			
6a- Was the standard reporting form provided for reporting suspected medication errors?			
6b- Was the standard reporting form provided for reporting suspected counterfeit/substandard medicines?			
6c- Was the standard reporting form provided for reporting therapeutic ineffectiveness?			
6d- Was the standard reporting form provided for reporting suspected misuse, abuse of and/or dependence on medicines?			
6e- Was the standard reporting form provided for reporting ADRs by the general public?			
7- Is there a process in place for collection, recording and analysis of ADR reports?			

8- Was pharmacovigilance incorporated into the the national curriculum of the various health care professions?			
8a- for medical doctors?			
8b- for dentists?			
8c- for pharmacists?			
8d- for nurses or midwives?			
8e- for others?<br>− to be specified;			
9- Is there a newsletter, information bulletin, and/or website as a tool for dissemination of information on pharmacovigilance?			
10- Is there a national ADR or pharmacovigilance advisory committee or an expert committee in the setting capable of providing advice on medicine safety?			

**Indicators**	**Answer**	**Description**
**Core process indicators**		
1- How many ADR reports were received by the center in 2017?<br>*Definition:* Valid case reports should contain the four core data elements, as per ICH-E2A:<br>1. Reporter<br>2. Identifiable patient<br>3. Suspected medicines<br>4. Adverse reaction.		
2- How many reports are there in the national database currently? (Since its inception to 2017)		

3- How many reports were acknowledged and/or issued feedback in 2017?		
4- How many reports were subjected to causality assessment in 2017?		
5- How many reports were satisfactorily completed and submitted to the national pharmacovigilance center in 2017?<br>*Definition:* Total reports received yearly at the pharmacovigilance center that have all the relevant fields for causality assessment satisfactorily filled in.		
5a- Of the reports satisfactorily completed and submitted to the national pharmacovigilance center, how many were submitted to the WHO database?		
6- How many reports of therapeutic ineffectiveness were received in 2017?<br>*Definition:* Failed treatments owing to the lack of effectiveness of medicines used in the healthcare system.		
7- How many reports on medication errors were reported in 2017?<br>*Definition:* Failure in treatment processes that resulted in harm to patients.		
8- How many registered pharmaceutical companies are there in Iran?		
9- How many registered pharmaceutical companies have a functional pharmacovigilance system?		
10- How many active surveillance activities were initiated, ongoing, or completed from 2013-2017?		

**Core process indicators**		
1- How many signals were detected from 2013 to 2017 by the pharmacovigilance center?		
2- How many regulatory actions were taken in 2017 consequent to national pharmacovigilance activities?		
2a-Product label changes (variation)?		
2b- Safety warnings on medicines?		
2c- Drug withdrawals?		
2d- Other restrictions on the use of medicines?		
3- How many people were admitted to the hospital as a result of events associated with medicines and their use in 2017?		
4- How many medicine-related deaths reported to the national pharmacovigilance center?		
**Pharmacovigilance indicators for public health programs (PHP)**		
1- There are pharmacovigilance activities in place within the PHP routinely? *Definition:* The presence or absence of key pharmacovigilance activities in the PHP: to report suspected ADRs to the pharmacovigilance center, using or adapting the standard ADR form recommended by the pharmacovigilance center; and to have an open communication link with the pharmacovigilance center, to analyze and react to drug-related problems.		
2- Was pharmacovigilance systematically considered in all the main treatment guidelines and protocols in use within the public health programme?		

3- Was the standard reporting form provided for reporting in settings?<br>3a- suspected medication errors?<br>3b- suspected counterfeit/substandard medicines?<br>3c- therapeutic ineffectiveness?<br>3d-suspected misuse, abuse of and/or dependence on medicines?		
4- How many ADR reports were collected within the public health programme in 2017?		
5- How many reports on therapeutic ineffectiveness were received from PHP in 2017?		
6- How many satisfactorily completed reports were submitted to the national pharmacovigilance center in 2017?		
6a- Of the reports satisfactorily completed and submitted to the national pharmacovigilance center, how many were submitted to the WHO database?		
7- How many patients were admitted to the hospital with a medicine-related illness attributable to a PHP's preventive or healing regimen during 2017?		
8- How many PHP medicine-related deaths reported to the national pharmacovigilance center in 2017?		
